# It Takes More than Two to Tango with COVID-19: Analyzing Argentina’s Early Pandemic Response (Jan 2020–April 2020)

**DOI:** 10.3390/ijerph18010073

**Published:** 2020-12-24

**Authors:** Analya Romo, Citlaly Ojeda-Galaviz

**Affiliations:** 1 College of Letters, Arts, and Sciences, University of Arizona, Tucson, AZ 85721, USA; 2 Global Health Institute, University of Geneva, 1205 Geneve, Switzerland; citlaly.ojeda@etu.unige.ch; 3 School of Social Work, Arizona State University, Phoenix, AZ 85006, USA; 4 College of Science, University of Arizona, Tucson, AZ 85721, USA

**Keywords:** COVID-19, transmission, health systems, disruption, PAHO, WHO, Argentina, pandemic, economic, SEIR, LSA, Buenos Aires

## Abstract

In November 2019, the world was introduced to a new coronavirus that has since ravaged it. Argentina began to see an increase of COVID-19 quickly in the new year and as of April 2020 the country was still being burdened by the transmission of the virus. With the progression of the epidemic turning into a pandemic, health authorities constantly updated health prevention strategies and responses to the novel coronavirus in its first wave. The Center for Disease Control and Prevention (CDC) issued a level three warning for international travel to/from Argentina because of COVID-19′s rapid transmission. With Argentina’s already fragile economy, health systems had to meet the challenge of being able to treat the infected. This case presentation aims to provide an overview of Argentina’s earliest epidemiological situation of the COVID-19 pandemic. The data provided in this study concern Argentina’s COVID-19 situation during the period of January 2020–April 2020. Mathematical modeling was used to forecast COVID-19 transmission after the first wave, specifically focusing on Buenos Aires. The country’s demographics and an impression of its health systems will be analyzed in this case presentation for preparedness. The case study concludes in depicting Argentina’s current and anticipated economic, social, and political disruptions because of the first wave of the pandemic.

## 1. Introduction

In November 2019, the first human cases of a respiratory infection from a novel strain of a Severe acute respiratory syndrome (SARS) were identified in Wuhan, China [1]. Clusters first appeared to be solely in China but as it continued to spread to neighboring countries; by the end of January 2020, the World Health Organization (WHO) declared a “Public Health Emergency of International Concern” [2]. The source of the virus and mode of transmission was still unknown around this time, but it did not deter the virus from spreading to more than 160 countries. Symptoms of the coronavirus varied between individuals but shared commonalities with “a presence of a fever and one or more respiratory symptoms” [3]. There is also the “unknown role and magnitude of asymptomatic cases; difficulty in identifying cases due to non-specific symptoms and possibility of co-circulation of other respiratory pathogens (e.g., influenza, respiratory syncytial virus [RSV]) hence potential for undetected transmission” [3].

This novel coronavirus shared similarities with previously encountered viruses such as: SARS (2002) or Middle Eastern Respiratory Syndrome (MERS) (2012) [4]. There are many types of human transmitted coronaviruses that cause “mild upper-respiratory tract illnesses” and due to this novel-coronavirus came from the same strain it was named COVID-19 [5]. The CO stands for corona, VI for virus and D for disease, and 2019 is for the year it was discovered [4].

The first case in Argentina was a 43-year-old man who recently returned from a trip from Milan, Italy, on 3 March 2020. Four days later Argentina had the first COVID-related death in Latin America [6]. On 11 March 2020, the WHO declared the COVID-19 outbreak a pandemic as the world experienced more than 100,000 cases, and the number of deaths was over 4200 [7]. Of those confirmed cases, 19 were in Argentina. The president of Argentina issued a national health emergency which was to be in effect for one year [6] in light of the WHO’s announcement. This decree was meant to begin non-pharmaceutical preventative measures to decrease the spread of virus. The decree also gave the ministry of health enforcement authority over further health measures. 

The arrival of this new pandemic could create problems for Argentina because its health systems and economy are not equipped to handle it. A pandemic has the power to overwhelm health systems and since Argentina has a fragile health system, it is plausible for it to collapse. Not only would this pandemic affect the health of individuals, but it would severely impact, “wide geographic areas and cause significant economic, social, and political disruption,” [8].

Throughout history, Argentina has experienced various forms of financial strains due to: poverty-related diseases, climate change, high incidences of HIV [9], and human rights issues. As Argentina attempts to heal from these previous instabilities, their progress may be lost if the transmission of COVID-19 continues to increase. 

As of April 2020, there were 2443 confirmed cases and 105 deaths in Argentina [6]. The country was on a lockdown that closed schools, borders and socially distanced many activities. The ministry of health has consistently updated daily reports on the situation and health recommendations throughout the nation to slow the spread of the virus. Argentina has also been collaborating to provide complete transparency over COVID-19 measures and health promotion as well. 

A real-time analysis was conducted as the pandemic was impacting Argentina in its first wave. This information will be relevant in discussing the country’s pandemic preparedness and the consequences of its evolving epidemiological situation. Mathematical modeling would also be used to predict future COVID-19 infections in Argentina, specifically in Buenos Aires to provide an example of a high-transmission region.

## 2. Case Presentation

### 2.1. Argentinian Context and Its Demographics

**Figure 1:**
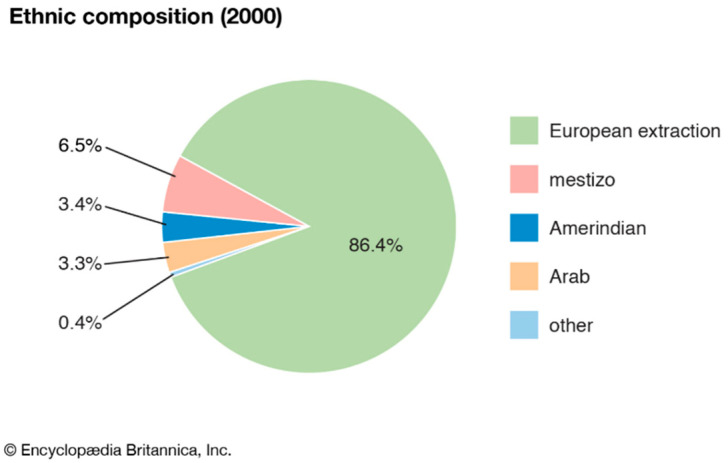
Ethnic composition in Argentina. [10].

Before Argentina’s colonization by Spain in the 16th century, this area was occupied solely by several diverse indigenous people [10]. These indigenous groups of people were forced into labor by the Spanish settlers and were all but exterminated throughout the colonial period. After three centuries, Argentina claimed its region which placed it between Uruguay and Chile in 1816. The majority of the ethnic composition of Argentina is from European descent (86.4%) and only 6.5% being mestizo (which is of indigenous descent) as can be seen in Figure 1 [10].

The variance in “natural resources, climate and topography shaped the patterns of European settlement,” [10]. Despite Argentina now being an integrated society, the country still follows “patterns that were determined in colonial times” [10]. Birth rates also declined in the early 20th century and are now among the lowest in the continent. This year, there are estimated to be 45,479,118 inhabitants in Argentina [11]. Around 24.02% of the population is under 15 years of age, 63.79% are between the ages of 15–64 and 12.13% are over 65. The majority of the population live in urban areas compared to rural areas. One-third of the population lives in the capital. Its largest cosmopolitan city is Buenos Aires and it has a similar size to Rome or Paris [11].

The South American country has the climatic characteristics of majorly temperate; arid in southeast and subantarctic in southwest [11]. It is the “world’s eighth largest country and is made up of: immense plains, deserts, tundra, mountains and thousands of miles of ocean coastline” [10]. These landscapes have served a great resource for Argentina for its agricultural activity.

Argentina has one of the largest economies in Latin America with a gross domestic product (GDP) of approximately 470 billion USD. Argentina is one of the largest exporters of beef products, the USDA reported that in 2019 Argentina would produce 575,000 tons, the highest volume since 2009, [12]. Aside from exporting beef, Argentina exports: soybeans, lemons, seeds, grapes, corn, tobacco, peanuts, tea, wheat and livestock. The main export partners are: Brazil (16.1%), USA (7.9%), China (7.5%), Chile, (4.4%) [11]. The agricultural sector accounts for 10.8% of the economy, the industrial sector for 28.1% and the service sector for the majority of 61.1%. 

The republic of Argentina holds a democratic political system, and its administrative divisions are split into 23 provinces [11]. The current president of Argentina was sworn into office in December of 2019. Argentina is and has been a member state of the United Nations as well as the Pandemic Influenza Preparedness framework (PIP) where member states benefit from data sharing and collaboration during a pandemic. 

Throughout history, Argentina has experienced various forms of obstacles which ranged from poverty-related diseases, recessions, climate change, high incidences of HIV [13] and human rights issues. These obstacles severely impact the well-being of the Argentinian population. These societal predispositions will influence how effective non-pharmaceutical interventions imposed by the Argentine government will determine the country’s pandemic preparedness. 

### 2.2. Health Care Systems

Argentinians life expectancy is 77.8 years of age and compared to other countries it is 74th on the list [11]. There are 16 births per 1000 inhabitants and the maternal mortality rate is 39 deaths per 100,000 lives births. Accessibility to healthcare and drinking water sources have improved since 2015 including in urban areas. The government extends 9.1% of funds to healthcare goods and services [11] which is below average, compared to the United States at 16.9% [11]. The 23 provinces of the country, “function independently and have constitutional responsibility for the leadership, financing and delivery of health services” [5].

Health systems in Argentina are composed of three different sectors: private, public, and social security. The public sector is encompassed by both national and provincial health ministries; thus including public hospitals that provide free health services. These particularly service low-income individuals, which comprise 36% of the population [14]. The social security sector is encompassed by national and provincial insurance that covers workers. The private sector is encompassed by private insurers to cover health professionals and private facilities [5]. This sector includes insurance companies with prepayments plans. Private insurance is only held by 8% of the population. 

Argentina has one of the most insubstantial health systems in all of the Americas and it would benefit from strong bureaucratic efforts to recruit stakeholders that share health objectives [15]. Currently, the health systems have objectives that focus on being able to guarantee universal health coverage for the entire nation. This objective may exist but has not been achieved this year. The three main reasons that Argentina’s health systems are fragile are: lack of medical coverage, regulatory functions and geographic disparities [5]. The common factor is the variance in developmental disparities between leadership and accessibility. 

Argentina’s health authority has been limited in “how effectively it can require provincial governments to adhere to new national legislation that involves structural change—given the resources it administers and the country’s federal structure” [5]. Normally, the main way to put into effect such changes is through wide unanimity. This had been attempted before through federal health plans and by strengthening the role of the Federal Health Council (COFESA) which has had varying outcomes in the past. 

There are 3.6 physicians and 3.2 hospital beds per 1000 inhabitants in the country overall, the specifics can vary between jurisdictions. Even with this availability, the Pan American Health Organization (PAHO) recognizes the “potential risks from new pathogens and zoonotic diseases, calling attention to the need to adapt and strengthen the surveillance system at all levels,” [16]. Before the pandemic, Argentina was already dealing with high rates of neglected tropical disease (NTD), non-communicable diseases (NCD) malnutrition and maternal mortality [15]. 

Overall, health systems in Argentina attempt to provide accessible care for all and even though it does invest more than other Latin American countries it is still not enough. It is difficult for people to afford premiums or have accessibility to attend health centers [5]. These complications could impact Argentina negatively if hit drastically with the novel pandemic. 

## 3. Findings

### 3.1. Epidemiological Situation

Argentina was one of the countries to react fairly quickly in regard to curbing infections. The Argentinian authorities were monitoring the situation closely and began to create necessary measures to prepare for the potential aftermath. The global health situation between 31 December 2019 to 28 February 2020 saw a total of 83,631 laboratory-confirmed cases of COVID-19, and 2858 deaths were reported from 51 countries, with many of the cases still being within China [17]. During this period of time the Hubei Province in China had accounted for the majority of COVID-19 cases and deaths [17]. However, clusters were beginning to materialize in Italy, Iran, Japan and the Republic of Korea due to travelers arriving from countries where community transmission was rapid [17]. Due to the unknown factors relating to asymptomatic cases these figures could be much higher than reported. 

**Figure 2:**
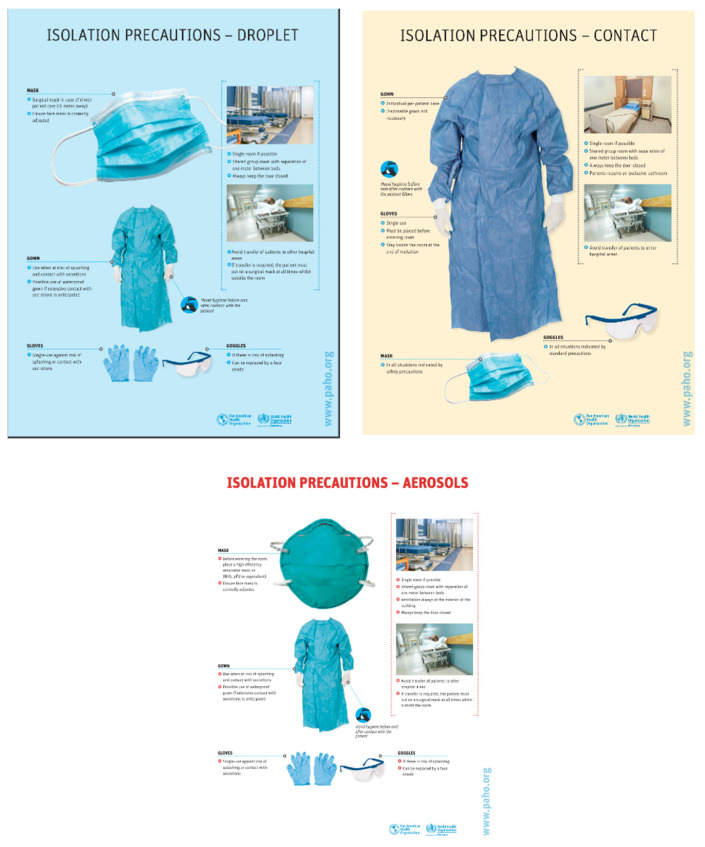
Communication materials provided to staff and medical settings to curb the spread of infection [18].

On 16 January 2020, it was recommended for infection prevention and control (IPC) to seek early recognition and control of the possible sources of infection in the hospital setting [18]. At the end of January, the PAHO/WHO continued renovating their recommendations for health systems and testing to be done throughout the Americas. Due to there not being a cure or vaccination for the virus, health care workers focused on the implementation of health services, as well as clinical management. Communications regarding health preventative measures in hospitals were reiterated to staff and were offered in Spanish, English and French [18]. The visuals (Figure 2) were easy to follow and manageable to place around clinical settings. 

Patients with suspected and/or confirmed symptoms were to be isolated in an individual room. All health facilities had to communicate to the public how much isolation space was being utilized and available. It was also necessary for health officials to follow up with any individual who had contact with an infected person. Although travel was not restricted, individuals arriving would be given information regarding COVID-19 and what procedures were in place to seek medical attention if symptoms arise, as well as hygienic precautions, [17]. On 14 February 2020, the majority of all state parties in the Americas, “have implemented complementary measures involving points of entry and international travelers. Examples of complementary measures include entry screening, public health observation and risk communication,” [17]. Prevention and early screening continued to be a priority in detecting COVID-19 in member states. On 11 March 2020, the WHO officially declared the COVID-19 outbreak an epidemic turned pandemic [16]. 

Due to this announcement in mid-March 2020, “Argentina stepped up measures to stem the spread of coronavirus, extending the health emergency for one year and imposing a flight ban originated from the most affected countries” [19]. On 20 March 2020, the ministry of health in Argentina ordered its citizens to remain in their homes starting at midnight. Citizens were to refrain from leaving homes until 31 March. However, the population was allowed to go outside for food, medication, and cleaning supplies [20]. Only a few people were exempt from said regulations and the military would be on the streets to enforce compliance and if not they would be sanctioned [19]. 

On 18 of March, the WHO began solidarity clinical trials of potential COVID-19 treatments and Argentina was among the member states included in this study [21]. This solidarity trial is meant to, “evaluate the effect of drugs on three important outcomes in COVID-19 patients: mortality, need for assisted ventilation and duration of hospital stay”, [22]. Results of this trial will be announced in late 2020. 

**Figure 3:**
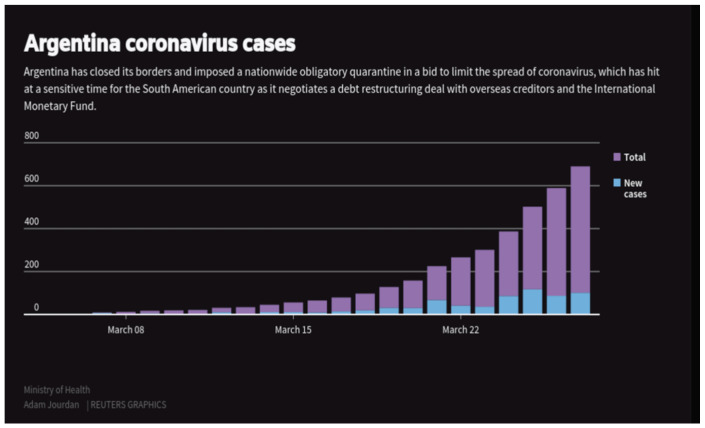
Graph of confirmed COVID-19 and new cases in Argentina from 8 March–22 March [23].

On 19 of March the 100th case of COVID-19 was confirmed in Argentina [23]. On 20 March 2020, the ministry of health distributed 57,000 COVID-19 tests to different provinces in Argentina [24]. The professionals who administered the tests were provided training by the National Administration of Laboratories and Health Institutes (ANLIS) and PAHO [23]. The training and the “diagnostic techniques recommended by the World Health Organization (WHO); SARS-CoV-2 detection protocols; the interpretation of results; performing exercises with clinical cases and reporting the results in the SISA” [25]. Results would be returned to individuals within 24–48 h of the test. On 25 March 2020 there were 502 confirmed cases along with 8 deaths and “the health ministry also confirmed for the first time that cases of local person-to-person ‘community transmission’ had been detected” [26]. On 22 March 2020 there were 41 new cases where 17 infected individuals traveled through a high zone of transmission, 11 were through exposure and 12 are still being investigated (Figure 3). This would bring Argentina’s total of confirmed COVID-19 cases to 266 with 4 deaths. 

The Center for Disease Control and Prevention (CDC), issued a level 3 warning on 27 March 2020, warning the public to not travel to Argentina because their medical facilities have limited resources [27]. If a traveler falls sick while traveling to Argentina, they were not guaranteed medical treatment if medical facilities were overwhelmed [27]. The promptness in distributing this information to the public highlights the country’s assertiveness when it comes to protecting the nation’s health systems, which is the foundation of the International Health Regulations. Argentina is working with the IHR in being transparent and accountable for the management and information of COVID-19 to ensure the safety and health of the nation. By the end of March, there were 649 cases where 54% of cases were imported and 25% from local exposure [12]. The president extended the nationwide lockdown to curb community transmission until 13 April [28].

**Figure 4:**
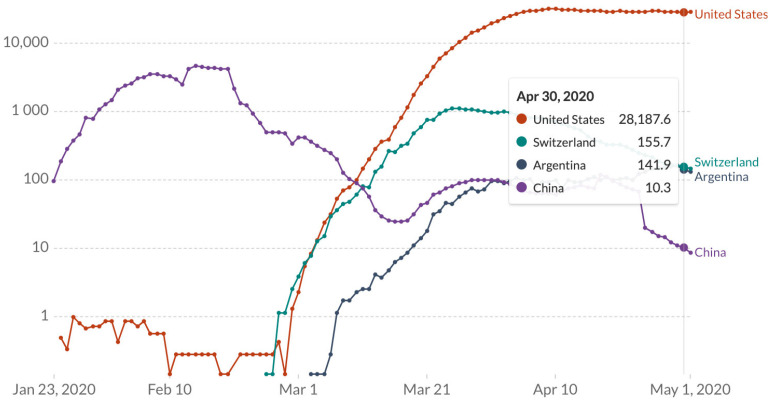
Scale of cumulative confirmed cases of COVID-19 in Argentina, Switzerland, China and the United States. Data retrieved from John Hopkins Coronavirus Resource Center. Last updated 1 May 2020.

According to the ministry of health, it is predicted that the first wave will peak in April or May 2020 [28]. The figure below does confirm there is a steady recline of confirmed cases in Argentina. As of 30 April 2020, there were 4438 cumulative confirmed cases in Argentina with the prevalence infection rate of 141.9 (Figure 4) cases per 1000 inhabitants [28]. This number could be higher but because of limited testing they are lower than actual case figures. Hospitals across Argentina created space for 179 new patients and intensive care unit (ICU) equipment to meet the positivity rate [29].

The other main factor for inaccuracies in confirmed COVID-19 cases is the underreporting of asymptomatic cases. People without symptoms are less likely to get tested. Health experts studying symptomatic and asymptomatic nursing home residents in the United States claim, “asymptomatic transmission of SARS-CoV-2 is the Achilles’ heel of Covid-19 pandemic control” [30] because it can be undetectable until tested. If asymptomatic individuals are not also being included in mass testing; the transmission of the virus will not cease.

### 3.2. Mathematical Modeling

**Figure 5:**
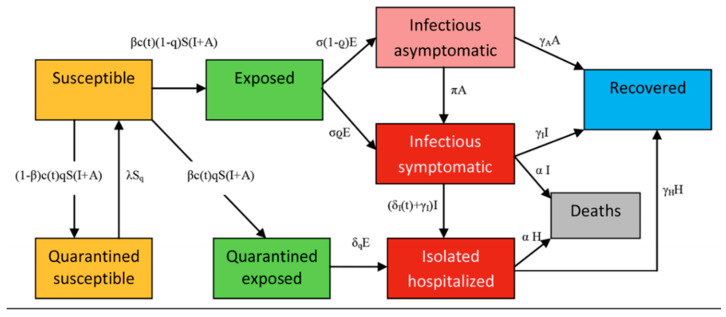
A simplified schematic diagram of the COVID-19 transmission model using SEIR model [32].

Epidemiological tools are used in order to monitor how effective preventative measures are in terms of an epidemic or in this case a pandemic. The (SEIR) model (susceptible, exposed, infectious, recovered) is used to determine the dynamics of transmission in COVID-19 outbreaks [31]. The figure (Figure 5 below shows the intensities of community transmission in terms of Argentina’s largest city, Buenos Aires. This city is a high-risk region in Argentina because of its large population of over 15 million people [32]. Not only is the population crowded in this particular region but there are a lot of underprivileged individuals who cannot isolate themselves, imposing a risk of contagion. 

**Figure 6:**
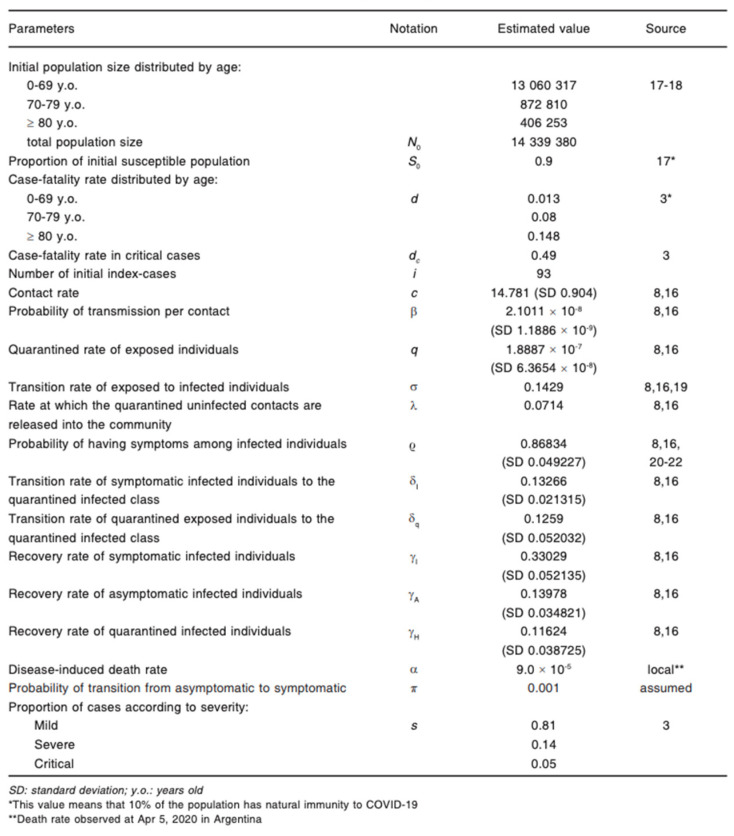
COVID-19 transmission forecasting using the SEIR compartmental model for Buenos Aires [33].

The following equations were used to compute values for Figure 6. These values were expressions of various non-pharmaceutical interventions, governmental interventions and health preventative strategies used during the beginning of the pandemic [33]. Data collected from the beginning of the pandemic (20 March 2020) using a 5-month forecast with the starting figure of 93 confirmed cases [33]. Different scenarios were used for comparing purposes such as: no interventions, low interventions and high interventions. The four assumptions used for each scenario were: “the whole population was susceptible, unprotected contact lead to infection, positive cases of asymptomatic COVID-19 were considered ‘non sick’” [33].

S’ = −(βc(t) + c(t)q(1 − β))S(I + A) + λSq (1)

E’ = βc(t)(1 − q)S(I + A) − σE(2)

I’ = σ E − (δI (t) + α + γI )I(3)

A’ = σ(1 − )E − γAA(4)

S’q = (1 − β)c(t)qS(I + A) − λSq(5)

E’q = βc(t)qS(I + A) − δq Eq(6)

R’ = γI I + γAA + γHH(7)

It is predicted that there would be a peak of 65,000 symptomatic infected people towards the end of August 2020 if there were no interventions [33]. Global health experts predict that a second wave would potentially hit Buenos Aires (or Argentina as whole) from October 2020–January 2021 [33]. Numerous outbreaks were likely to occur almost immediately when lockdown lifts. If the inputs of the model are changed to a “larger quarantining population, the infection curves shifted right until October–December 2020 [33]. 

**Figure 7:**
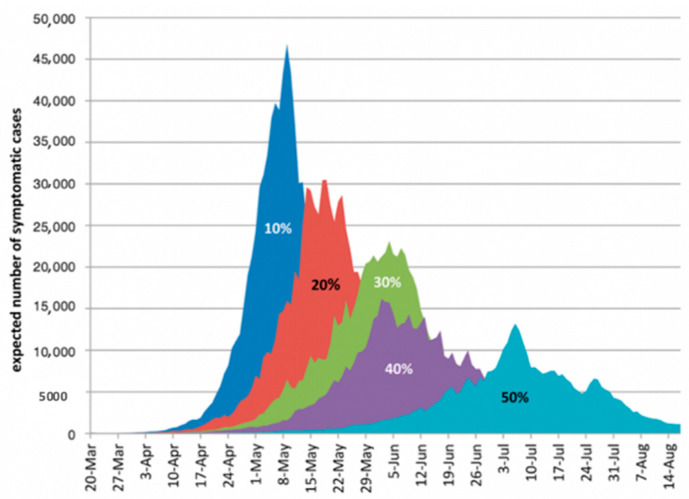
Expected symptomatic COVID-19 cases in a 5-month quarantine period.

If the population was quarantined properly and took all health precautions the curve would flatten in the 5 month period (Figure 7). Infection rates would completely decrease if the number of quarantined individuals increases [33]. 

**Figure 8:**
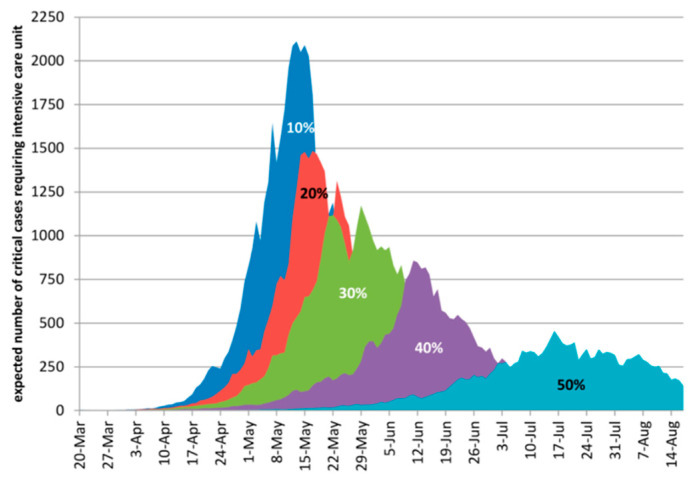
Expected cumulative COVID-19 cases requiring intensive care in Buenos Aires (according to different forms of intervention) [33].

Figure 8 provides a visual representation of how many COVID-19 patient’s intensive care. In this scenario there would be peaks of: 2111, 1486, 1173, 858 and 456 patients (percentage portrays the population in quarantine) [33]. 

**Figure 9:**
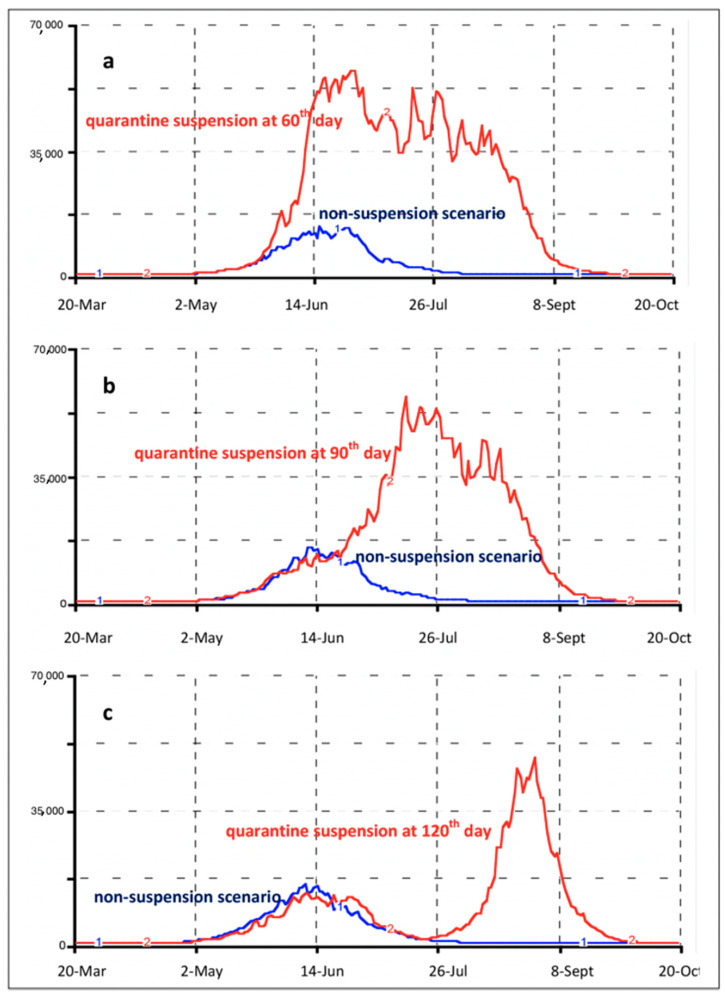
Graphics depicting the effects of interrupted quarantine in 60–90–120-day intervals in Buenos Aires using 40% of a quarantined population [33].

Figure 9 provides a visual for the effects of interrupted quarantine in Buenos Aires. This has a *p*-value of 0.40 and in intervals of 60–90–120 days. Each scenario shows that cases would increase, and a short quarantine period would only delay another peak in infections [33]. This is due to susceptible citizens leaving isolation after a sudden interruption of the quarantine at either the 60- or 90-day mark. These results are comparable with the 120-day mark having a 30-day rebound in reinfection [33]. 

These graphics depict how strong health preventative measures can flatten the curve and can decrease infection rates not just in Buenos Aires but in Argentina as a whole. Flattening the curve would help the nation’s health and budget medical supplies that are already overwhelmed with the pandemic. It is also worth mentioning that asymptomatic individuals must be included in mathematical modelling because they contribute to community transmission. Asymptomatic individuals are currently at 17.9–50% of the population and are increasing [33]. These graphics also prove that abandoning restrictions could mean recurring COVID-19 which can cause a second or even third wave. Quarantine goes hand in hand with non-pharmaceutical interventions in order to decrease the spread of the virus. This data can support policies and health authorities when strategically planning exit strategies. 

## 4. Management and Outcomes

### 4.1. Non-Pharmaceutical Interventions

**Figure 10:**
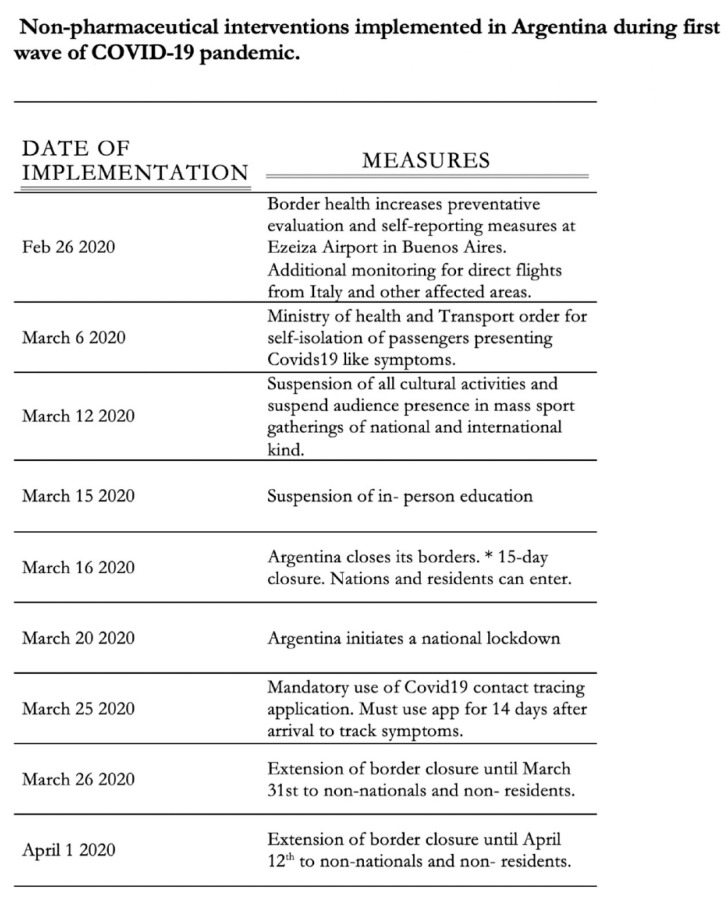
Timeline of non-pharmaceutical interventions [34].

The Ministry of Health in Argentina provided and created various prevention practices for the general population based on WHO measures. Initially the ministry of health was evaluating and creating safety measures by surveying airports with main connections to highly affected countries. As shown in our figure (Figure 10), the non-pharmaceutical interventions began on 26 February 2020 with the control measures of direct flights from Italy to the main Buenos Aires airport. March brought on the most impactful and obvious changes to Argentina lifestyle. Subsequently, in March border restrictions were introduced and gradually extended as numbers continued to increase on national and international scale [34].

**Figure 11:**
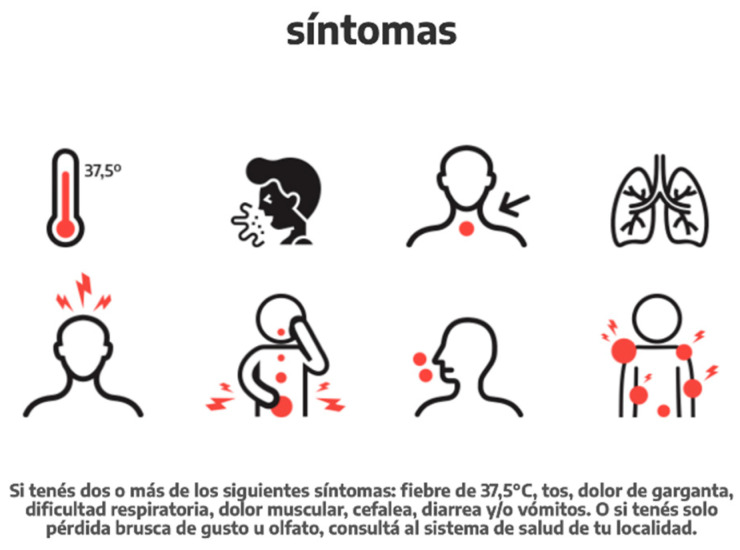
Infographic of COVID-19 symptoms provided by the national government [37].

**Figure 12:**
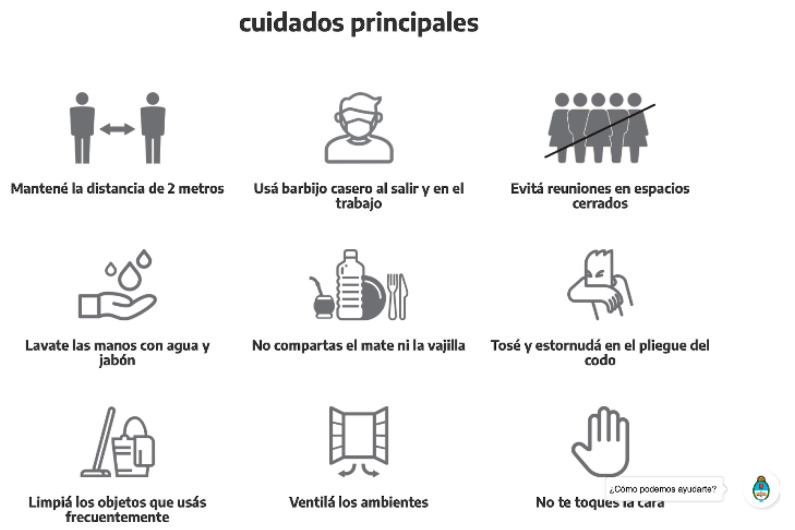
Infographic of COVID-19prevention measures for Argentina’s public [37].

The Argentine population was asked to track their symptoms and the creation of a symptom tracking app for new arrivals with obligatory utilization for 14 days upon arrival. Figure 11 illustrates an infographic designed by the government to help identify symptoms and recommend self-isolation in case of symptom appearance [34]. Within this time frame, national museums were closed as well as other public/cultural activities in order to decrease rates of infection [35]. Finally, the Argentinian government encouraged daily prevention methods such as hand washing, distancing, proper mask wearing, and discouraged gatherings in confined spaces (Figure 12). Alongside a daily layer of preclusion and self-monitoring system, the nation went into a national lockdown on 20 March. The lockdown did not allow civilians to leave their homes with the exception of buying groceries and medicine [36] and online education was encouraged. 

### 4.2. Economic Impact-Observed v. Expected

Before the announcement of the pandemic, Argentina was experiencing a serious and long-lasting economic crisis. The nation displayed close to double-digit unemployment rates, an accumulating debt of 57 million dollars, and a 50% annual inflation [6]. In 2019, Argentina inaugurated president Alberto Fernandez as Argentina entered its third year of recession. Early into Alberto Fernandez’ presidency, he introduced an economic package that intended to alleviate Argentina’s economic crisis. Debt payments and the restructuring of Argentina’s financial stability has been setback due to the health crisis [34]. Experts warn that if this plan is postponed much longer it is at risk of failing which would only further the country’s default [2]. 

In order to assist the population economically and help implore the financial state of the nation, various economic plans were proposed throughout the first wave of the pandemic. The first was introduced on 10 March, the Argentinian government created a fund of 1.7 million pesos to fortify its economic plan in preparation of the hit of the pandemic. This economic prevention plan was meant to purchase laboratory and hospital equipment [38]. Additionally, one of the efforts imposed by the Fernandez administration was to freeze prices on food products to remain as they were in early March, before the official lockdown [29].

The nationwide lockdown was mandatory and only essential workers continued operations normally. The president promoted, “health first, then economy” [2] in order to not only encourage the public to stay in their homes while also showing that their health is being prioritized. This quarantine was extended many times during the first wave and there were rising concerns “about how long the quarantine can last before people start to suffer from lost income, job losses and rising debt levels” [2]. The unemployment rate stayed consistently at 8.9% from last year’s fourth quarter [2]. The poverty rate from 2019 has already risen to 35.4% and is projected to increase as COVID-19 creates economic setbacks [2]. 

The government proposed a stimulus package of 11 billion in order to, “sustain economic activity, avert food and medical supply shortages, help companies and protect workers and vulnerable groups affected by the crisis” [2]. Another package would provide relief to small business, self-employed workers and low-income households [2]. The president also appealed to employers to not lay off workers which would keep unemployment rates from increasing [2]. 

There was a positive trend in e-commerce through the first wave “from 24 February to 22 March, MercadoLibre Inc, the region’s largest e-commerce marketplace experienced a 28% increase in newly registered customers—equating to 1.7 million people” [29]. This trend resulted because many-if not all-stores were closed throughout the nation. 

The ministry of economics introduced a package of measures to protect production and employment, control prices, and guarantee essential supplies [39]. An extra measure of protection was added to the emergency family income (IFE) which assisted citizens with a higher degree of vulnerability between the ages of 18 to 65 years old. The minister of economics stated that there currently three measures (direct money transfers, job protection, and unemployment insurance) that can be taken to cope with the world economic crisis that COVID-19 will create, and Argentina was the only country adopting all three models [40]. 

Despite the various economic preventions from the national government, experts anticipate a devastating economic impact from COVID-19. Argentina is a predominantly agricultural country and carries the title of highest income producing country based on their exports [41]. Currently, Argentina provides soybean products to countries that have been affected largely by COVID-19. For example, Italy is its 3rd highest consumer, followed by Spain and the United States [34]. Economically, Argentina is one of the strongest exporters in South America of soybean products but is currently facing a recession. However, it is estimated that exports will drop by 3% due to COVID-19 [42]. If this prediction is accurate, it would be another colossal loss to the country and “if the lockdown extends for longer than 14 April, there are concerns of a deeper recession,” [2]. 

### 4.3. Social and Political Disruption

COVID-19 has universally caused a social and political disruption for many countries and their citizens. Without fail Argentina is experiencing the same effects. Following COVID-19′s pandemic status, Argentina closed its borders for non-residents for 30 days and temporarily stopped issuing visas to citizens of the United States, UK, China, Japan, South Korea, and other European countries, and many Argentinian citizens were initially stranded [26]. Later, methods of return were introduced and precautions for those reentering the country were implemented. 

For the citizens that have remained within Argentina’s borders, schools and national parks closed on 31 March. [43]. The extension of border closure continued until April and schools will remain on virtual platforms even after the projected end of the lockdown [44]. Political action was creating various social disruptions beyond the initial panic. President Fernandez first announced the national lockdown on 20 March and explained that the mandatory quarantine would begin in a slow and segmented form [45]. While the introduction promised to be relaxed, the consequences of violation were not. Any violation of quarantine would result in a fee of 200,000 pesos, monies collected from fees will be utilized to fund the healthcare system [32]. The national government also attempted to purchase food provisions for highly vulnerable populations and imposed laws to delay unemployment. However, food prices were non-negotiable, and President Fernandez canceled the assistance [44]. Due to this failure, the supportive packages mentioned previously were created.

**Figure 13:**
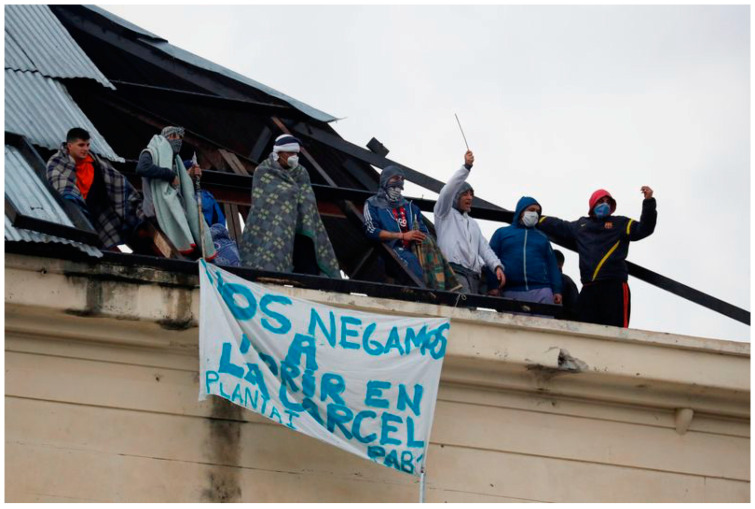
Argentinian prisoners protesting rising COVID-19 rates from prison overcrowding [46].

Even with national support a pandemic has the power to overwhelm healthcare systems and Argentina’s is fragile. With the number of cases and increasing death count, 123 laboratories opened around the nation for increased testing [44]. Additionally, shortages of preventive supplies quickly became another issue as citizens began panic buying masks in pharmacies, the president encouraged people to make home-made masks to alleviate the scarcity [44]. However, restrictions and preventions are still not enough for some of the most vulnerable groups. Overcrowding has increased numbers in prisons and prompted officials to consider house arrest for some inmates to decrease the high COVID-19 rates within prisons. However, thousands of citizens have begun protesting from home against their release (Figure 13). Feminist groups and relatives of victims are advocating against the release of those convicted of sex crimes [29]. On the other hand, prisoners have also begun protesting and set the prison on fire. Some created banners that read “We refuse to die in jail” [46]. Heightened civil unrest and panic around COVID-19′s uncertainty are trickling into various aspects of Argentina’s social life. 

To alleviate COVID-19 related panic, the Ministry of Justice released recommendations by Investigations of the National Institute against Discrimination, Xenophobia, and Racism (INADI). The INADI findings express how to deal with and perceive coronavirus to avoid misinformation in the media and social media as well as avoid racism, xenophobia, and general panic [47]. The ministries’ recommendations varied from refraining from calling COVID-19 “ the Chinese virus” and abstaining from making apocalyptic comparisons [47].

Argentina’s multifaceted approach to COVID-19 prevention and assistance strives to create the least amount of disruption in the social realm. These approaches are supported by the quick political reaction of the government through its ministries. However, it is through these same responses that the government has created the most social shift in daily Argentinian life. The current lockdown is constantly extending at a social and financial cost.

## 5. Discussion

As the global situation worsens under the grasp of COVID-19, Argentina is attempting to save its nation from an irreversible aftermath. This stressful situation has strained the social, political and economic sectors and it is important for Argentina’s national authorities to support its citizens during this time. Argentina was one of the first countries to impose a nationwide lockdown that was extended several times throughout the first wave. This lockdown was intended to curb community transmission. Extending the lockdown would only increase economic setbacks although it was admirable that the approach of the Argentinian government prioritized public health over the economic setbacks that were occurring simultaneously [2]. The calculations from the mathematical modelling supported a longer isolation period along with other non-pharmaceutical methods [34]. This would help curve the infection rate and decrease the possibility of reinfection.

Despite being one of the strongest economic presences in South America, Argentina is in its 3rd year of recession; this would in turn delay economic regrowth. The government attempted to provide food provisions to low-income families, but the purchase was ultimately unsuccessful due to overpayment [44]. This left communities hungry and struggling to make ends meet. If the nation is not eating nutritious food, they will have decreased immune health which would in turn make them susceptible to the virus. Not only were low-income families not receiving adequate nutrition, there was a lot of panic buying throughout the nation. On 2 March 2020, masks sold out across pharmacies, [48]. This occurred before the nation had one confirmed case. In response to this the president encouraged citizens to make their own home-made masks if needed [4]. This was a great response to the issue at hand because it is low-cost, and it is a more sustainable way to mask up.

The increase of COVID-19 deaths in Argentina encouraged health authorities to provide more testing sites. This increased the testing sites to 123 laboratories [44]. This may increase accessibility, but it still does not account for asymptomatic individuals. These asymptomatic individuals still contribute to community transmission. A suggestion to also minimize the communal spread is to implement contract tracing. The government focused mainly on quarantining and social distancing. Contract tracing could also help narrow down the origin of the transmission and extend testing. This could also differentiate the symptomatic and asymptomatic as well.

Argentina’s health systems are attempting to use all of their resources and healthcare staff available to meet the needs of the COVID-19. Thus far, the country has created extensive and informative materials to educate and raise awareness of prevention methods against COVID-19 [35]. Hospitals increased space for COVID-19 patients as well as increasing ICU units [29]. This was an example of Argentina using the resources at hand to make do and meet the demands of the pandemic.

Even though Argentina is one of the most resilient countries in Latin America it did not demonstrate pandemic preparedness during COVID-19′s initial phase. Health systems were overwhelmed with infected patients and the country was deep in a stagnant recession due to a delayed financial restructuring. The disruptions caused by the virus will not only worsen Argentina’s recession but could potentially cause an international recession. As officials from the Argentine Chamber of Commerce and Services, the Argentine Industrial Union (UIA) and the CGT, decide on a post-COVID economic plan, “there was consensus across the government about the need for a gradual exit from mandatory lockdown” [44]. The restrictions did lift gradually in April in certain service industries [49], but will it be enough to save Argentina’s economy? Government officials plan a soft reopening of the nation later this year with a 20 billion peso relief package to aid workers with “training, credits and the purchase of machinery” [50]. As the unpredictable pandemic continues, the outcome is debatable.

## 6. Conclusions

Accepting the peril, a late response could bring, Argentina was one of the nations that acted speedily by curbing infections as prompt as possible. Even though the nation acted and attempted to utilize all its resources, their healthcare system remained fragile and deterred authorities from full pandemic preparedness. In an attempt to aid these systematic shortcomings, various ministries attempted to provide political support in hopes of decreasing infection rates and minimizing social disruption. Political involvement gave the country great assistance in the sense of improved testing accessibility and basic needs accessibility [50]. However, these policies also caused much civil unrest through ever-expanding lockdowns and mismanagement of highly vulnerable populations [46].

Statistical predictions, if correct, do not promise that Argentina will be unmarked by the pandemic. The government has taken the lead on economic assistance through a triple assistance method whilst attempting to strengthen a weak economy. Agriculture is the main source of income for this country and COVID-19 projects a decrease in exports while complying with IHR agreements and FAO regulations [51].

The impacts of the COVID-19 global pandemic are constantly evolving and affecting all nations as of the end of April. With precautions and statistical projections, it is still difficult to predict the outcome of this pandemic. We suggest further studies are conducted once this pandemic ends and the impacts left on Argentina can be fully investigated. Areas of interest can be the economic, social, and medical interventions beyond this time frame. Given the uncertainty, the first wave of the pandemic has proven to be fatiguing all sectors in Argentina.

Using the SEIR tool is popular in epidemiological situations because it provides a logistical forecast of the effects of a pandemic. This calculated data from the mathematical modelling could serve as a decision-making tool for health authorities for the future of the nation. Furthermore, predictions such as these also help the country prepare systematically and economically for the hard years that Argentina is sure to face after the COVID-19 virus has stabilized. Argentina has exhibited resilience even before COVID-19 devastated the globe, and with increased preparedness it can overcome it.

## Data Availability

Publicly available datasets were analyzed in this study. This data can be found here: [http://medicinabuenosaires.com/revistas/vol80-20/destacado/original_7162.pdf].
